# Persistent inequities in complete denture rehabilitation among older adults in Brazil between 2010 and 2023

**DOI:** 10.1590/1980-549720260029.supl.1

**Published:** 2026-07-31

**Authors:** Herbert Lucas Nascimento Gomes, Sophia Queiroz Marques dos Santos, Angelo Giuseppe Roncalli, Tamires Carneiro de Oliveira Mendes

**Affiliations:** I Universidade Federal do Rio Grande do Norte – Natal (RN), Brazil.

**Keywords:** aged, health inequities, complete denture, oral health, health surveys

## Abstract

**Objective:**

To analyze the evolution of inequalities in the distribution of complete denture use and need among older adults in Brazil, considering socioeconomic factors in 2010 and 2023.

**Methods:**

An analytical study was conducted using data from the Brazilian National Oral Health Surveys SB Brasil 2010 and 2023, with representative samples of individuals aged 65 to 74 years. The outcome integrated information on complete denture use and need, distinguishing need due to edentulism or replacement. Independent variables included sociodemographic and socioeconomic characteristics, with emphasis on the intersectional analysis of gender, race/skin color, and education. Descriptive analyses and the Concentration Index (CI) were performed to measure inequalities, accounting for the complex sampling design.

**Results:**

A slight increase was observed in the proportion of individuals who do not use and do not need dentures, particularly in the South (from 3.8%; 95% confidence interval — 95%CI 2.1–6.8 to 9.1%; 95%CI 5.7–14.3) and Southeast (from 2.3%; 95%CI 1.2–4.2 to 13.9%; 95%CI 9.1–20.7) regions. Nevertheless, around 90% of older adults still required complete dentures, either due to edentulism or replacement needs. Black men with low education concentrated the highest frequencies of need due to edentulism and showed the smallest reduction over the period. Concentration curves revealed the persistence of inequalities according to gender, race/skin color, education, and income.

**Conclusion:**

Despite advances in public oral health policies, improvements in denture use and need occurred unevenly, favoring socially advantaged groups and maintaining gaps in oral health care among older adults in situations of greater vulnerability.

## INTRODUCTION

Inequalities in oral health are driven by underlying social determinants, including the conditions in which individuals are born, grow, live, work, and age, as well as structural factors such as the unequal distribution of power, wealth, and resources within society^1^. From an intersectional perspective, the interplay among characteristics such as age, gender, and race/skin color is recognized, with these factors mutually influencing one another and shaping social positions that affect life experiences^2,3^. Consequently, health experiences are influenced by interpersonal and structural systems of oppression, including racism, sexism, and ageism, which perpetuate processes of social exclusion and inequities among this population, the fastest-growing demographic group in Brazil and worldwide^4^.

The Brazilian Institute of Geography and Statistics (*Instituto Brasileiro de Geografia e Estatística* – IBGE) estimates that, by 2070, older adults will account for 37.8% of the Brazilian population, resulting in substantial impacts across social, economic, governmental, labor, and family domains^5,6^. Population aging, together with social, economic, and urban transformations, has altered patterns of living, working, and eating, thereby contributing to the epidemiological transition^7^.

In this context, oral diseases are among the most prevalent health conditions, and tooth loss represents one of their most significant and enduring consequences^1,8^. Edentulism has historically been a common characteristic among older adults, with aging recognized as an important risk factor. In Brazil, these conditions are monitored through national oral health epidemiological surveys, particularly the SB Brasil survey, which enables the monitoring of trends in various oral health conditions and supports the planning and evaluation of oral health policies and interventions^9^. According to SB Brasil 2023, 36.48% of individuals aged 65 to 74 years exhibit complete tooth loss, a prevalence higher than the global average of 23%^1^. To address this issue, the National Oral Health Policy (*Política Nacional de Saúde Bucal* – PNSB) reoriented the care model to expand access to prosthetic rehabilitation within the public health system through Primary Health Care services, Dental Specialty Centers (*Centros de Especialidades Odontológicas* – CEOs), and Regional Dental Prosthesis Laboratories (*Laboratórios Regionais de Prótese Dentária* – LRPDs)^10^.

Therefore, prosthetic rehabilitation represents one of the primary components of oral health care for older adults and is currently used by approximately 50.15% of this population. However, 53.2% of older adults still require some form of prosthetic rehabilitation, either because of edentulism or the need for prosthesis replacement^5^. In cases requiring replacement, previous access to rehabilitation services is evident; however, a new prosthesis is needed due to functional impairment or the risk of oral lesions. In contrast, prosthetic need resulting from edentulism reflects a more severe condition, characterized by limited access to care and adverse effects on nutrition, social interaction, and quality of life^8,11,12^.

Despite the importance of distinguishing between different types of prosthetic needs, previous studies have generally addressed this issue in a simplified manner, without adequately considering variations in severity across conditions or identifying the groups most vulnerable to each situation. Therefore, this study aimed to analyze trends in inequalities in the distribution of prosthesis use and prosthetic need among older adults from different socioeconomic strata between 2010 and 2023.

## METHODS

This study was based on the two most recent national oral health surveys, known as the SB Brasil 2010 and SB Brasil 2023 projects, both coordinated and funded by the Ministry of Health. These surveys had nationwide coverage and regional representativeness. They were household-based studies in which oral examinations were conducted and questionnaires were administered to assess oral health conditions, socioeconomic characteristics, use of dental services, and self-perceived oral health. Both surveys employed similar and comparable methodologies with respect to sampling design, age groups, and the oral health conditions investigated^9,13^. Accordingly, the use of variables collected through comparable procedures was intended to minimize measurement bias and enhance data comparability.

For this study, a sample of older adults aged 65 to 74 years was selected. The initial sample comprised 7,619 individuals in 2010 and 9,745 individuals in 2023, including those requiring complete dentures for the analyses. This age group was chosen to assess the cumulative effects of public policies over the life course and their ability to address complex oral health conditions, such as the need for extensive prosthetic rehabilitation.

This article was prepared in accordance with the Strengthening the Reporting of Observational Studies in Epidemiology (STROBE) guidelines^14^.

### Variables

In both surveys, complete denture rehabilitation was assessed using two variables: denture use and denture need, each evaluated separately for the maxillary and mandibular arches. In addition, denture quality was assessed using the Prosthesis Quality Index. A limitation of these measures is their inability to determine whether the identified need resulted from an edentulous individual without a denture or from the presence of a denture requiring replacement.

Thus, the primary outcome was constructed by combining three variables:

a) Edentulism: Based on the “missing” component of the Decayed, Missing, and Filled Teeth (DMFT) index, a variable was created with the categories “maxillary edentulism,” “mandibular edentulism,” and “edentulism in both arches.”
b) Denture use: The original variables were recategorized to generate a single variable with the categories “use of a complete maxillary denture,” “use of a complete mandibular denture,” and “use of complete dentures in both arches.”
c) Need for dentures: Similarly, the original variables were combined to create a new variable with the categories “need for a complete maxillary denture,” “need for a complete mandibular denture,” and “need for complete dentures in both arches.”

These three variables were combined to create an outcome variable that initially comprised a large number of categories. The variable was subsequently reclassified into the following categories:

1. Does not use and does not require a denture in either arch: individual who is not edentulous in either arch.
2. Rehabilitated with complete dentures in both arches: individual who is edentulous in both arches and uses dentures in satisfactory condition, with no need for replacement.
3. Requires a complete maxillary and/or mandibular denture replacement: individual who is edentulous in one or both arches and uses one or two dentures that require replacement.
4. Requires a complete maxillary or mandibular denture due to edentulism: individual who is edentulous in at least one arch, does not use a denture, and therefore requires prosthetic rehabilitation.
5. Requires complete dentures in both arches due to edentulism: individual who is edentulous in both arches, does not use dentures, and requires complete prosthetic rehabilitation.

In this categorization, the first category represents the most favorable condition, whereas the last corresponds to the least favorable outcome. From a health planning perspective, the first category reflects a population with no need for specialized prosthetic services. The second category includes individuals with oral sequelae who have undergone rehabilitation and possess an adequate prosthesis. The remaining three categories comprise individuals requiring prosthetic rehabilitation services, with varying levels of treatment need.

Accordingly, this dependent variable was considered to have an ordinal structure. For standardization purposes, it was designated as “complete denture use and need.”

The individual-level independent variables were selected based on the use of comparable data collection procedures across both surveys, thereby ensuring data comparability. Accordingly, the sociodemographic variables “gender” and “race/skin color,” as well as the socioeconomic variables “income” and “educational status,” were included.

The variable “gender” was retained with the categories “male” and “female.” Self-reported race/skin color was recategorized as “White” and “Black” (comprising individuals categorize as “Black” or “Brown”), consistent with the classification adopted in SB Brasil based on criteria established by IBGE^9,13^. Individuals classified as “Yellow” and “Indigenous” were excluded because of their low frequency, the difficulty of allocating them to the study groups, and the potential for bias associated with self-reported classification. The “Yellow” category represented 1.6% (n=121) of the sample in 2010 and 1.3% (n=129) in 2023, whereas the “Indigenous” category represented 0.9% (n=72) in 2010 and 0.5% (n=50) in 2023. Therefore, the low frequency of these groups could introduce substantial limitations to the external validity of findings related to these population segments. In the present study, race/skin color was treated as a social marker and interpreted as an expression of historical and structural processes of inequality.

“Family income” corresponded to the total income received by all household members during the month preceding the survey. In 2023, this variable was recorded as a continuous value in Brazilian reais (R$), whereas in 2010 it was categorized into seven income brackets (from “up to R$ 250” to “R$ 9,500 or more”). To ensure comparability between surveys, income was converted into multiples of the minimum wage based on the official value in effect at the time of each survey. According to data from the Brazilian government, the minimum wage was R$ 510 in 2010 and R$ 1,320 in 2023^15^. This standardization enabled greater comparability between the datasets. Subsequently, income was categorized into four groups: “less than 1 MW,” “between 1 and 2 MW,” “between 3 and 5 MW,” and “more than 5 MW.”

“Educational status” was assessed as years of formal schooling (0 to 16 years) using the same methodology in both surveys and was therefore retained in its original form. For selected analyses, the variable was dichotomized according to the median value observed in 2023, resulting in the categories “low educational status” (<8 years of schooling) and “high educational status” (≥8 years of schooling). For the period during which this age group (65 to 74 years of age) had access to formal education, these categories correspond, respectively, to incomplete primary education and complete primary education or higher.

To assess the effects of intersectionality, an analysis was conducted using a combined variable derived from “gender,” “race/skin color,” and “educational status.” The inclusion of “income” in this composite measure was also tested; however, it resulted in a considerable number of missing values.

The combination of these three dichotomous variables resulted in a composite variable with eight categories. These categories were analyzed in relation to the worst outcome to assess their distribution and identify a gradient of inequality, according to the following order (from the least favorable to the most favorable condition):

1. Black man with low education;
2. Black woman with low education;
3. White man with low education;
4. Black man with high education;
5. Black man with high education;
6. Black woman with high education;
7. White man with high education;
8. White woman with high education.

This classification was established *a priori*, based on the assumption that, in the context of complete denture needs among older adults, males, Black race/skin color, and lower educational status constitute important exposure factors. This ranking is specific to the outcome examined in the present study and should not be extrapolated to other contexts or variables.

Finally, the regions of Brazil (North, Northeast, Central-West, Southeast, and South) were included as a contextual variable, as they reflect differences in socioeconomic conditions, demographic characteristics, and the organization of health services^16^.

### Statistical analysis

Descriptive analyses were conducted based on the proportional distribution of the outcomes according to the combinations of the dependent and independent variables. In addition, region was included as a contextual variable to identify regional differences. All analyses were performed using data from 2010 and 2023.

To measure inequality in the distribution of the outcome, the Concentration Index (CI) was used. This index is calculated in a manner similar to the Gini coefficient and is based on the Lorenz curve^17^. The CI compares the cumulative proportional distribution of a socioeconomic variable with the cumulative proportional distribution of a given outcome. A value of zero (CI=0) indicates perfect equality and is represented by a straight line, whereas values ranging from −1 to 1 indicate different degrees of inequality, with the extreme values representing the maximum level of inequality. Because the outcome categories were ordered from the most favorable to the least favorable condition and the exposure variables from the least favorable to the most favorable condition, negative CI values indicate inequality^18^.

In all analyses, the complex sampling design was taken into account, including sampling weights and design effects, for the estimation of parameters and the calculation of CI. For the latter, the Stata package developed by the International Center for Health Equity at Universidade Federal de Pelotas was used and is available at https://equidade.org/pt/home.

### Data availability statement

The authors used publicly available data from the SB Brasil surveys to conduct this study. The dataset is available at: https://www.gov.br/saude/pt-br/composicao/saps/brasil-sorridente/sb-brasil/dados

## RESULTS

The final sample comprised 4,462 individuals in 2010 and 5,222 in 2023. As described in the Methods section, individuals classified as “Yellow” and “Indigenous” in terms of race/skin color were excluded. In addition, as only complete denture needs were considered, individuals with other rehabilitation needs, such as partial dentures, were not included in the analysis.

The descriptive analysis of the outcome variable in relation to the contextual variable (region) and the individual-level variables is presented in Tables 1 to 3, with percentage estimates and corresponding confidence intervals for each study year. The analysis of prosthesis use and need among older Brazilian adults shows important changes between 2010 and 2023 and highlights persistent patterns of regional, racial/ethnic, and socioeconomic inequalities.

**Table 1:** Distribution of frequencies and corresponding confidence intervals for the need for complete dentures among older adults (65 to 74 years), according to year and region. Brazil, 2025.

	Year	2010		2023	
		n	Est. (95%CI)	n	Est. (95%CI)
North	Does not use and does not need a prosthesis in either arch	14	1.2 (0.5–2.5)	51	3.5 (2.1–5.8)
	Rehabilitated with CD in both arches	224	18.6 (14.3–23.7)	274	22.3 (16.5–29.3)
	Needs CD in upper and/or lower arch due to replacement	488	42.2 (37.2–47.4)	629	44.0 (37–51.4)
	Needs CD in upper or lower arch due to edentulism	275	23.0 (18.2–28.6)	205	20.1 (16.5–24.2)
	Needs CD in both arches due to edentulism	129	15.0 (11.5–19.4)	117	10.0 (7.5–13.3)
Northeast	Does not use and does not need a prosthesis in either arch	29	1.6 (0.9–2.6)	89	4.0 (2.1–7.5)
	Rehabilitated with CD in both arches	316	17.9 (14.9–21.3)	276	13.1 (9.6–17.5)
	Needs CD in upper and/or lower arch due to replacement	480	44 (39.1–49)	814	47.4 (41.9–53.0)
	Needs CD in upper or lower arch due to edentulism	268	21.7 (18.4–25.4)	327	22.2 (18.7–26.2)
	Needs CD in both arches due to edentulism	162	14.9 (11.5–19)	190	13.3 (9.8–17.8)
Southeast	Does not use and does not need a prosthesis in either arch	43	2.3 (1.2–4.2)	128	13.9 (9.1–20.7)
	Rehabilitated with CD in both arches	243	31.9 (24.3–40.7)	144	18.3 (13.7–24.0)
	Needs CD in upper and/or lower arch due to replacement	295	38.6 (30.9–46.9)	365	49.7 (43.2–56.2)
	Needs CD in upper or lower arch due to edentulism	115	15.2 (10.5–21.4)	94	10.5 (7.3–14.9)
	Needs CD in both arches due to edentulism	70	12.1 (8.2–17.3)	52	7.6 (4.5–12.5)
South	Does not use and does not need a prosthesis in either arch	37	3.8 (2.1–6.8)	95	9.1 (5.7–14.3)
	Rehabilitated with CD in both arches	233	40.6 (32.5–49.1)	198	33.6 (27.4–40.4)
	Needs CD in upper and/or lower arch due to replacement	205	36.5 (26.7–47.5)	291	43.9 (36–52.0)
	Needs CD in upper or lower arch due to edentulism	85	14.2 (9.7–20.3)	78	8.3 (5.5–12.5)
	Needs CD in both arches due to edentulism	35	5.0 (2.8–8.7)	30	5.1 (2.8–9.3)
C. West	Does not use and does not need a prosthesis in either arch	17	1.7 (0.8–3.3)	60	6.8 (4.5–10.3)
	Rehabilitated with CD in both arches	178	22.8 (17.6–28.9)	139	18.6 (12.9–26.1)
	Needs CD in upper and/or lower arch due to replacement	338	44.5 (37.5–51.7)	417	56.2 (48.7–63.3)
	Needs CD in upper or lower arch due to edentulism	113	18.4 (14.9–22.5)	97	12 (8.8–16.1)
	Needs CD in both arches due to edentulism	70	12.7 (8.7–18.2)	62	6.4 (4.1–10)
Brazil	Does not use and does not need a prosthesis in either arch	140	11.9 (7.2–19.0)	423	88.1 (81.0–92.8)
	Rehabilitated with CD in both arches	1,194	46.2 (37.8–54.8)	1,031	53.8 (45.2–62.2)
	Needs CD in upper and/or lower arch due to replacement	1,806	30.9 (24.5–38.0)	2,516	69.1 (62.0–75.5)
	Needs CD in upper or lower arch due to edentulism	856	39.1 (31.2–47.7)	801	60.9 (52.3–68.8)
	Needs CD in both arches due to edentulism	466	41.7 (31.8–52.2)	451	58.3 (47.8–68.2)

CD: Compete Dentures; 95%CI: 95% Confidence Interval; Est.: Estimate; n: Unweighted sample size.

Source: SB Brasil 2010 and 2023 data.

**Table 2:** Distribution of frequencies and corresponding confidence intervals for the need for complete dentures among older adults (65 to 74 years), according to year and sociodemographic and economic variables. Brazil, 2025.

Characteristic	2010				2023			
	n	Est. (%)	95%CI		n	Est. (%)	95%CI	
			LL	UL			LL	UL
Gender								
Male								
Does not use and does not need a prosthesis in either arch	58	3.5	2.1	5.9	161	9.6	6.6	13.8
Rehabilitated with CD in both arches	399	26.2	20.9	32.4	388	19.8	15.6	24.8
Needs CD in upper and/or lower arch due to replacement	560	36.4	29.4	44.0	852	45.8	40.5	51.1
Needs CD in upper or lower arch due to edentulism	311	16.6	11.5	23.4	307	13.4	10.7	16.7
Needs CD in both arches due to edentulism	230	17.3	12.9	22.8	236	11.5	8.6	15.1
Female								
Does not use and does not need a prosthesis in either arch	82	1.7	1.0	2.9	262	9.4	6.7	13.1
Rehabilitated with CD in both arches	795	33.1	27.0	39.8	643	19.4	16.0	23.3
Needs CD in upper and/or lower arch due to replacement	1,246	41.0	35.0	47.4	1,664	49.9	45.2	54.6
Needs CD in upper or lower arch due to edentulism	545	16.1	12.8	20.0	494	14.2	11.7	17.0
Needs CD in both arches due to edentulism	236	8.1	5.8	11.2	215	7.1	5.1	9.8
Self-reported Race/skin color								
White								
Does not use and does not need a prosthesis in either arch	99	3.6	2.3	5.6	236	15.0	10.5	21.1
Rehabilitated with CD in both arches	624	31.7	25.2	39.1	431	23.7	19.7	28.4
Needs CD in upper and/or lower arch due to replacement	813	43.3	36.3	50.7	867	46.6	42.0	51.3
Needs CD in upper or lower arch due to edentulism	326	14.7	11.3	18.9	219	9.6	7.0	13.0
Needs CD in both arches due to edentulism	145	6.7	4.8	9.2	113	5.0	3.3	7.5
Black								
Does not use and does not need a prosthesis in either arch	40	0.9	0.5	1.5	181	4.7	3.3	6.6
Rehabilitated with CD in both arches	537	28.3	22.2	35.2	572	15.8	13.0	19.2
Needs CD in upper and/or lower arch due to replacement	938	35.1	28.4	42.5	1,555	49.3	44.5	54.2
Needs CD in upper or lower arch due to edentulism	512	18.4	13.7	24.2	551	17.8	15.2	20.8
Needs CD in both arches due to edentulism	305	17.4	12.6	23.5	324	12.3	9.2	16.3
Education (years of schooling)								
8 years or more								
Does not use and does not need a prosthesis in either arch	98	8.6	4.9	14.8	340	21.1	16.3	26.8
Rehabilitated with CD in both arches	266	32.7	24.0	42.7	496	20.1	16.5	24.2
Needs CD in upper and/or lower arch due to replacement	376	47.5	36.8	58.5	1,040	46.4	41.6	51.4
Needs CD in upper or lower arch due to edentulism	94	6.4	4.2	9.7	222	9.0	6.5	12.4
Needs CD in both arches due to edentulism	41	4.8	2.0	10.8	89	3.4	2.1	5.4
Up to 7 years								
Does not use and does not need a prosthesis in either arch	39	1.0	0.5	2.1	69	2.0	1.1	3.7
Rehabilitated with CD in both arches	900	30.2	24.6	36.5	515	19.9	16.4	23.8
Needs CD in upper and/or lower arch due to replacement	1,385	38.3	32.7	44.3	1,422	50.3	45.3	55.2
Needs CD in upper or lower arch due to edentulism	735	18.3	14.4	23.0	550	16.8	14.2	19.8
Needs CD in both arches due to edentulism	401	12.1	9.3	15.7	332	11.0	8.4	14.4
Monthly family income								
3 or more MW								
Does not use and does not need a prosthesis in either arch	96	5.2	2.8	9.4	169	29.3	19.5	41.4
Rehabilitated with CD in both arches	384	38.4	30.0	47.6	155	22.2	16.2	29.5
Needs CD in upper and/or lower arch due to replacement	489	40.7	31.6	50.6	259	42.7	32.7	53.4
Needs CD in upper or lower arch due to edentulism	160	7.2	4.8	10.8	49	3.9	2.1	7.2
Needs CD in both arches due to edentulism	66	8.4	4.8	14.3	25	1.9	0.8	4.3
Up to 2 MW								
Does not use and does not need a prosthesis in either arch	41	1.4	0.7	2.6	140	5.8	3.8	8.7
Rehabilitated with CD in both arches	771	28.3	22.9	34.3	566	19.5	15.9	23.8
Needs CD in upper and/or lower arch due to replacement	1,249	39.0	33.2	45.1	1,558	47.8	43.3	52.3
Needs CD in upper or lower arch due to edentulism	666	19.0	15.2	23.3	548	16.7	13.8	20.0
Needs CD in both arches due to edentulism	387	12.4	9.4	16.3	275	10.2	7.8	13.3

CD: Complete Dentures; MW: Minimum wage; 95%CI: 95% Confidence Interval; Est.: Estimate; LL: Lower Limit; UL: Upper Limit.

Source: SB Brasil 2010 and 2023 data.

**Table 3:** Frequency distribution and corresponding confidence intervals for the need for complete dentures in older adults (65 to 74 years), according to year and intersectional categories (gender, race/skin color, and education). Brazil, 2025.

Characteristic		Black man with low education	Black woman with low education	White man with low education	Black man with high education	White woman with low education	Black woman with high education	White man with high education	White woman with high education
Does not use and does not need a prosthesis in either arch	Est. (%)	1.0	0.2	2.0	7.0	1.4	1.3	13.1	9.7
	95%CI	(0.4–2.4)	(0.01–0.50)	(0.8–5.0)	(2.6–18.0)	(0.4–5.0)	(0.5–3.3)	(5.2–29.4)	(5.5–16.3)
	n	13	8	8	9	10	8	27	53
Rehabilitated with CD in both arches	Est. (%)	24.2	32.9	23.2	26.8	32.9	15.3	33.4	41.6
	95%CI	(15.5–35.8)	(25.6–41.1)	(16.8–31.2)	(12.5–48.6)	(24.2–42.8)	(7.2–29.8)	(19.7–50.7)	(30.2–54.0)
	n	143	293	130	35	308	51	70	104
Needs CD in upper and/or lower arch due to replacement	Est. (%)	32.0	32.7	40.1	41.8	45.9	67.9	41.8	44.6
	95%CI	(21.8–44.3)	(25.6–40.7)	(30.4–50.6)	(22.3–64.3)	(35.9–56.2)	(46.1–84)	(26.4–59.1)	(32.2–57.7)
	n	215	515	190	58	422	121	63	126
Needs CD in upper or lower arch due to edentulism	Est. (%)	19.2	20.5	19.1	7.3	16.2	7.9	9.2	3.0
	95%CI	(9.8–34.3)	(15.1–27.3)	(12.6–27.9)	(3.4–14.8)	(11.5–22.2)	(3.5–16.9)	(4.8–16.8)	(0.9–9.3)
	n	160	291	95	21	178	25	21	24
Needs CD in both arches due to edentulism	Est. (%)	23.6	13.7	15.5	17.0	3.8	7.50	2.50	1.20
	95%CI	(15.2–34.6)	(9.6–19.3)	(10.3–22.6)	(4.8–45.7)	(2.2–6.3)	(1.1–36.7)	(0.8–7.5)	(0.5–3)
	n	121	143	68	16	55	7	9	8
2023									
Does not use and does not need a prosthesis in either arch	Est. (%)	1.1	1.4	2.2	10.3	3.5	12.3	28.5	29.6
	95%CI	(0.5–2.2)	(0.6–3.1)	(0.7–6.4)	(6.5–16)	(1.2–9.7)	(7.5–19.6)	(18.6–41.2)	(21.2–39.8)
	n	14	31	8	52	15	76	83	124
Rehabilitated with CD in both arches	Est. (%)	14.0	16.3	22.9	20.6	26.5	16.1	25.5	20.3
	95%CI	(9.1–21.2)	(11.8–22.1)	(14.4–34.3)	(13.6–29.9)	(20.1–34.1)	(11.3–22.3)	(16.1–37.9)	(12.8–30.6)
	n	107	194	66	104	129	157	92	134
Needs CD in upper and/or lower arch due to replacement	Est. (%)	45.7	49.2	55.9	48.4	51.9	59.6	34.5	41.4
	95%CI	(37.1–54.5)	(42.0–56.4)	(42.9–68.2)	(40.0–56.9)	(44.0–59.8)	(49.7–68.7)	(25.6–44.6)	(33.1–50.2)
	n	316	585	146	208	325	417	133	249
Needs CD in upper or lower arch due to edentulism	Est. (%)	19.5	21.1	9.9	14.3	13.3	9.2	7.2	6.9
	95%CI	(14.0–26.6)	(16.6–26.5)	(5.6–17.0)	(9.6–20.9)	(8.8–19.7)	(6.1–13.7)	(2.2–21.5)	(4–11.6)
	n	146	244	50	55	89	81	32	45
Needs CD in both arches due to edentulism	Est. (%)	19.7	12.0	9.0	6.4	4.7	2.9	4.3	1.8
	95%CI	(14.1–26.9)	(7.7–18.2)	(4.3–17.9)	(2.9–13.4)	(2.6–8.4)	(1.4–5.6)	(1.4–12.4)	(0.8–4.1)
	n	137	111	36	27	35	27	17	18

CD: Complete Dentures; 95%CI: 95% Confidence Interval; Est.: Estimate.

Source: Based on SB Brasil 2010 and 2023 data.

Between 2010 and 2023, there was an improvement in the oral rehabilitation conditions of older Brazilian adults, with a slight increase in the proportion of individuals who neither use nor require dentures, particularly in the South and Southeast regions. In 2010, individuals requiring some form of complete denture replacement predominated, while the proportion of those with no prosthetic need was low (up to 3.8%). In 2023, although the need for replacement dentures remained predominant, a modest improvement in the most favorable outcome (up to 13.9%) and a reduction in the need for dentures due to edentulism were observed. Despite these changes, the proportion of individuals with any prosthetic need remained around 90%.

The inequalities observed in the analyses according to gender, race/skin color, education, and income persisted throughout the study period. Women showed greater improvement in dental preservation, whereas men maintained a higher prevalence of unmet needs, despite a greater reduction in needs related to edentulism. Regarding race/skin color, improvements were observed across all groups; however, Black older adults remained at a disadvantage, with a lower proportion of individuals free from prosthetic needs in both years and a smaller reduction in needs due to edentulism. These findings reflect inequalities in both the quality and durability of prosthetic rehabilitation and in access to services.

Income and educational attainment were directly associated, with lower levels of education and income corresponding to poorer oral health indicators. In 2023, 29.3% of older adults in the higher-income group did not require dentures, compared with only 5.8% among those in the lower-income group. Despite improvements compared with 2010, social inequality appears to outweigh the positive effects achieved. Thus, although the overall scenario indicates progress, the findings show that improvements were not homogeneous, a pattern that becomes even more evident from an intersectional perspective.

An intersectional analysis of prosthesis use and need indicates a worsening of social and racial inequalities between 2010 and 2023. The less favorable outcome became more concentrated among older adults with cumulative characteristics of lower educational status, male gender, and non-White race/skin color, suggesting that progress resulting from policies aimed at oral rehabilitation has not been sufficient to reduce historical inequities. Structural barriers to access and care appear to have persisted, reinforcing existing patterns of social exclusion in the field.

**Figure 1:**
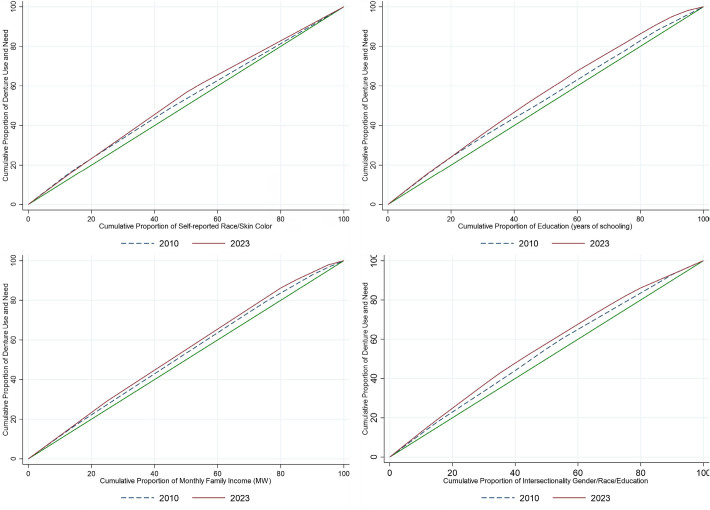
Concentration curves for denture use and need in relation to sociodemographic and socioeconomic variables. The green line represents the line of perfect equality of the concentration curves. Brazil, 2025.

Source: Based on SB Brasil 2010 and 2023 data.

**Figure 2:**
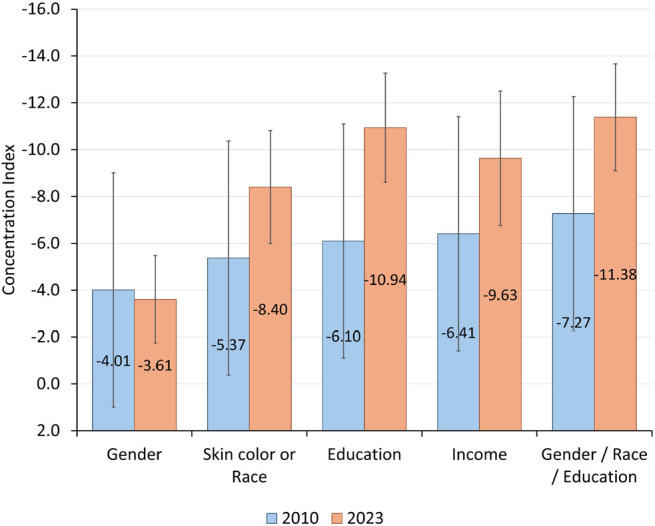
Values and corresponding confidence intervals for the Concentration Index for denture use and need, according to the independent variables used. Brazil, 2025.

Source: Based on SB Brasil 2010 and 2023 data.

Figure 1 presents the concentration curves (Lorenz curves) for prosthesis use and need according to four selected variables, and Figure 2 shows the CIs and their respective confidence intervals for the studied variables by year. The curves indicate that, between 2010 and 2023, income, education, race/skin color, and the intersectionality variable remained central determinants of inequalities, with a worsening pattern over the period.

Although relative improvements were observed in some groups, the curves and CI values show that the least favorable outcome remains concentrated among older adults in greater social vulnerability. Inequalities associated with income and educational status showed the highest levels, indicating that access to and quality of prosthetic rehabilitation continue to be strongly conditioned by structural factors. When gender, race/skin color, and education are combined, the index reaches its highest values, reinforcing the intersectional dimension.

These findings indicate that advances in public policies for oral rehabilitation and the improvements observed across sociodemographic groups have occurred unevenly, with better indicators concentrated among socially advantaged groups and persistent gaps in care among socially vulnerable older adults.

## DISCUSSION

The observed progress has not been homogeneous, with individual-level inequalities maintained or, in some cases, exacerbated. The reduction in tooth loss and access to prosthetic rehabilitation remain conditioned by socially structured factors. Thus, despite improvements in oral health, inequalities related to income, race/skin color, and educational attainment reflect a pro-wealthy pattern in access, which may be influenced by a still limited articulation between macro- and micro-level policies^19,20^.

Regional factors such as gender, race/skin color, educational status, and income continue to be associated with access to dental rehabilitation, reflecting challenges related to prior contact with preventive and health promotion actions. This context is consistent with the persistence of structural processes that organize access to health services, including structural racism^21^, which may have a greater impact on the older adults analyzed, who experienced life stages without a universal health system and, consequently, greater exposure to the social gradient in health.

The increased proportion of older adults without prosthetic needs among individuals with higher income and educational attainment may indicate limitations in oral health policies in promoting equity, as well as potentially reflecting greater access to private services, a pattern more frequent in the South and Southeast regions^5^, which concentrate more favorable socioeconomic conditions. This distribution is consistent with the concept of access, according to which the use of restorative dental services depends not only on availability but also on social factors that shape their utilization.

The analysis of these determinants from an intersectional perspective proved relevant, as the overlap of social markers produced a gradient of inequality, with different combinations associated with worse outcomes. These findings suggest that the effects of inequalities do not operate in isolation but rather accumulate. Although causality cannot be established, these patterns corroborate findings from other studies that indicate the persistence of structural barriers in access to oral health services and poorer health conditions among specific population groups^22-26^.

The findings indicate a lower proportion of Black individuals who are rehabilitated or do not require prostheses, and a higher prevalence of prosthetic replacement needs and edentulism. These results highlight inequalities in the distribution of the outcome, which, although associated with socioeconomic factors, are not exclusively explained by them, indicating complex disparities that extend beyond the health system. In this study, the race/skin color variable is understood as a social marker that reflects historical and structural processes of inequality. Such differences are related to the context of structural inequalities, including racism^26–28^.

Conversely, comparative analyses between 2003 and 2010 indicate more substantial improvements during a period marked by the expansion of social policies, which may have interacted with the observed outcomes, suggesting a possible dose-response relationship.

The expansion of primary care coverage and the consolidation of PNSB were fundamental to broadening access to dental services, including actions delivered by oral health teams (OHT) and specialized care provided by CEOs and LRPDs^29^. However, these initiatives remain insufficient to reduce health inequities, as the persistent need for prostheses among approximately 70% of older adults, whether due to edentulism or replacement, indicates that prosthetic rehabilitation remains a form of unmet demand^5,30^.

Inequities in the need for dental replacement prostheses and edentulism highlight structural inequalities related to access, quality, and continuity of dental care^31^. In this sense, it is necessary to move beyond strategies focused solely on expanding service supply, integrating rehabilitation, health promotion, and prevention actions aligned with the social determinants of the health-disease process. This requires sustainable priorities, adequate funding, and the strengthening of Primary Care as the organizing axis of the oral health care network, with a view to expanding equitable and universal access^32,33^.

Limitations include the possibility that the accuracy of the CI may have been partially affected by the use of an ordinal variable; however, the CI clearly identified patterns of inequality, highlighting its discriminatory capacity. The reduction of complex intersectional categories to an ordinal scale limits the approximation of the quantification of social dimensions and their interactions in real-world settings, indicating the need for further studies using more in-depth analytical strategies. The cross-sectional design of SB Brasil limits the analyses to the identification of associations, precluding causal inferences. Regarding Yellow and Indigenous individuals, the low representation of these categories in this important segment of the Brazilian population justifies their exclusion for greater parsimony in the statistical model. Strengths of the study include the use of a complex sampling design, with preservation of weights and representativeness, as well as the methodological innovation represented by the construction of a composite dependent variable incorporating different dimensions related to prosthetic need.

Despite improvements observed in the oral health of the older adult population, a high demand for replacement of inadequate prostheses persists. Addressing this problem requires, in addition to expanding coverage, the implementation of intersectoral public policies oriented toward territorial, racial/ethnic, and social equity. To this end, it is essential to strengthen the articulation between micro- and macro-level policies in order to promote health care that takes social vulnerabilities into account.
