# Supplementary material for: Persistent inequities in complete denture rehabilitation among older adults in Brazil between 2010 and 2023
Source: Rev Bras Epidemiol. 2026 Jul 31;29(Suppl 1):e260029supl1. doi: 10.1590/1980-549720260029.supl.1 (PMC13427309; doi:10.1590/1980-549720260029.supl.1)
Supplement: Translation

# Translation: Iniquidades persistentes na reabilitação por prótese total em pessoas idosas no Brasil entre 2010 e 2023

**DOI:** 10.1590/1980-549720260029.supl.1.2

**Authors:** Herbert Lucas Nascimento Gomes, Sophia Queiroz Marques dos Santos, Angelo Giuseppe Roncalli, Tamires Carneiro de Oliveira Mendes

**Affiliations:** I Universidade Federal do Rio Grande do Norte – Natal (RN), Brasil.

**Keywords:** pessoa idosa, iniquidades em saúde, prótese total, saúde bucal, inquéritos de saúde

## Abstract

**Objetivo:**

Analisar a evolução das desigualdades na distribuição do uso e necessidade de prótese total entre pessoas idosas brasileiras, considerando fatores socioeconômicos nos anos de 2010 e 2023.

**Métodos:**

Estudo analítico com dados dos inquéritos nacionais de saúde bucal SB Brasil 2010 e 2023, com amostras representativas de indivíduos de 65 a 74 anos. O desfecho integrou informações sobre uso e necessidade de prótese total, distinguindo necessidade por edentulismo ou substituição. As variáveis independentes incluíram características sociodemográficas e socioeconômicas, com ênfase na análise interseccional entre sexo, raça/cor e escolaridade. Foram realizadas análises descritivas e cálculo do Índice de Concentração (IC) para mensurar desigualdades, considerando o desenho amostral complexo.

**Resultados:**

Observou-se aumento discreto na proporção de indivíduos que não usam e não necessitam de prótese dentária, especialmente nas Regiões Sul (de 3,8%; intervalo de confiança de 95% — IC95% 2,1–6,8 para 9,1%; IC95% 5,7–14,3) e Sudeste (de 2,3%; IC95% 1,2–4,2 para 13,9%; IC95% 9,1–20,7). Ainda assim, cerca de 90% das pessoas idosas apresentavam necessidade de prótese total, fosse por edentulismo, fosse por substituição. Homens negros com baixa escolaridade concentraram maiores frequências de necessidade por edentulismo e menor redução no período. As curvas de concentração revelaram a persistência de desigualdades segundo sexo, raça/cor, escolaridade e renda.

**Conclusão:**

Apesar dos avanços nas políticas públicas de reabilitação bucal, as melhorias no uso e a necessidade de prótese ocorreram de forma desigual, favorecendo grupos socialmente favorecidos e mantendo lacunas no cuidado bucal de pessoas idosas em situação de maior vulnerabilidade.

## INTRODUÇÃO

As desigualdades em saúde bucal têm como determinantes sociais subjacentes as condições em que as pessoas nascem, crescem, vivem, trabalham e envelhecem, além dos fatores estruturais, como a distribuição desigual de poder, dinheiro e recursos na sociedade^1^. Sob a ótica da interseccionalidade, observa-se a confluência de características como idade, gênero e raça/cor, que se influenciam mutuamente, estabelecendo posições sociais que moldam as experiências de vida^2,3^. Assim, as experiências, como a saúde, são influenciadas por sistemas interpessoais e estruturais de opressão, como racismo, sexismo e idadismo, que reproduzem processos de exclusão social e iniquidades dessa população, que representa a que mais cresce no Brasil e no mundo^4^.

De acordo com o Instituto Brasileiro de Geografia e Estatística (IBGE), estima-se que, até 2070, as pessoas idosas representarão 37,8% da população brasileira, o que trará significativos impactos nas esferas social, econômica, governamental, trabalhista e familiar^5,6^. Com o envelhecimento populacional e as mudanças sociais, econômicas e urbanas, o modo de viver, de trabalhar e de se alimentar da população mudou, contribuindo para a transição epidemiológica^7^.

Nesse contexto, as doenças bucais se destacam como as mais comuns, e a perda dentária é uma de suas consequências mais expressivas e duradouras^1,8^. O edentulismo compõe um retrato histórico da pessoa idosa, sendo o envelhecimento um fator de risco. No Brasil, essas condições são monitoradas por inquéritos epidemiológicos nacionais em saúde bucal, especialmente o SB Brasil, que permite acompanhar tendências dos diversos agravos bucais, além de possibilitar o planejamento e a avaliação das ações em saúde bucal^9^. Segundo o SB Brasil 2023, 36,48% da população de 65 a 74 anos apresenta perda total de dentes, percentual superior à média global de 23%^1^. Para enfrentar este problema, a Política Nacional de Saúde Bucal (PNSB) reorientou o modelo de atenção, oportunizando o acesso à reabilitação protética na rede pública por meio da Atenção Primária à Saúde, dos Centros de Especialidades Odontológicas (CEOs) e dos Laboratórios Regionais de Próteses Dentárias (LRPDs)^10^.

Assim, a reabilitação protética é um dos principais eixos da atenção à saúde bucal da pessoa idosa, sendo utilizada por cerca de 50,15% dessa população. Entretanto, 53,2% desses indivíduos ainda necessitam de algum tipo de prótese, seja por edentulismo, seja por demanda de substituição^5^. Nos casos de substituição, observa-se que houve acesso prévio ao serviço, mas com necessidade de nova reabilitação em razão da perda de funcionalidade ou risco de lesões bucais. Já a necessidade por edentulismo reflete maior gravidade, marcada por dificuldades de acesso e impactos negativos na nutrição, convivência social e qualidade de vida^8,11,12^.

Apesar da importância de distinguir os tipos de necessidade de prótese, os estudos, em geral, têm-na considerado de forma sintética, sem ponderar as diferenças na gravidade de cada situação e quais seriam os grupos mais vulneráveis. Diante disso, o presente estudo teve como objetivo analisar a evolução da desigualdade na distribuição do uso e necessidade de prótese entre pessoas idosas de diferentes estratos socioeconômicos nos anos de 2010 e 2023.

## MÉTODOS

Este estudo fundamenta-se nos dois mais recentes inquéritos nacionais de saúde bucal, conhecidos como Projetos SB Brasil 2010 e 2023, coordenados e financiados pelo Ministério da Saúde, com abrangência nacional e representatividade regional. Trata-se de inquéritos de base domiciliar, nos quais foram realizados exames bucais e aplicados questionários para avaliar agravos bucais, condições socioeconômicas, utilização de serviços odontológicos e autopercepção de saúde bucal. Ambos os levantamentos utilizaram metodologia similar e comparável quanto ao plano amostral, aos grupos etários e aos agravos investigados^9,13^. Assim, a utilização de variáveis coletadas de forma semelhante buscou reduzir vieses de mensuração e garantir a comparabilidade dos dados.

Neste estudo, foi realizado um recorte da amostra, composto da população idosa de 65 a 74 anos. A amostra inicial incluiu 7.619 indivíduos em 2010 e 9.745 em 2023, sendo incluídos na análise aqueles que necessitavam de prótese total. A escolha dessa faixa etária ocorreu pela necessidade de entender os impactos das políticas públicas ao longo da vida e suas respostas a situações complexas, como a necessidade de grandes reabilitações.

O presente artigo foi elaborado de acordo com as diretrizes do checklist *Strengthening the Reporting of Observational Studies in Epidemiology* (STROBE)^14^.

### Variáveis

Em ambos os levantamentos, a reabilitação por prótese total foi avaliada por meio de duas variáveis: uso e necessidade de prótese, reportadas separadamente em ambos os casos para as arcadas superior e inferior. Adicionalmente, a qualidade da prótese foi avaliada com base no Índice de Qualidade de Prótese. Uma limitação dessas medidas é a impossibilidade de avaliar se a necessidade decorre de um indivíduo edêntulo que não usava prótese ou da presença de uma prótese que necessitava substituição.

Desse modo, o desfecho principal foi construído por meio da combinação de três variáveis:

a) Edentulismo: com base no componente “perdido” do índice de dentes cariados, perdidos e obturados (CPO-D), foi criada uma variável com as categorias “edêntulo superior”, “edêntulo inferior” e “edêntulo em ambas as arcadas”.
b) Uso de prótese: as variáveis originais foram recategorizadas, gerando uma única variável com as categorias “uso de prótese total superior”, “uso de prótese total inferior” e “uso de prótese total em ambas as arcadas”.
c) Necessidade de prótese: de modo similar, as variáveis foram combinadas, gerando uma nova variável com as categorias “necessidade de prótese total superior”, “necessidade de prótese total inferior” e “necessidade de prótese total em ambas as arcadas”

Essas três variáveis foram combinadas, gerando uma variável de desfecho inicialmente com grande número de categorias. Posteriormente, foi realizado um rearranjo, resultando nas seguintes categorias:

1. Não usa e não necessita prótese em nenhuma arcada: indivíduo não edêntulo em nenhuma das arcadas.
2. Reabilitado com prótese total em ambas as arcadas: indivíduo edêntulo em ambas as arcadas, que utiliza prótese em bom estado, sem necessidade de substituição.
3. Necessita prótese total superior e/ou inferior por substituição: indivíduo edêntulo em uma ou em ambas as arcadas, que utiliza uma ou duas próteses que necessitam ser substituídas.
4. Necessita de prótese total superior ou inferior por edentulismo: indivíduo edêntulo em alguma das arcadas, não usa e, portanto, necessita de uma prótese.
5. Necessita de prótese total em ambas as arcadas por edentulismo: indivíduo edêntulo em ambas as arcadas que não utiliza prótese e necessita de reabilitação total.

Nessa categorização, a primeira situação é a mais desejável, enquanto a última representa o pior desfecho. Para o planejamento em saúde, a primeira indica uma população sem necessidade de serviços protéticos especializados. A segunda indica indivíduos com sequela, porém reabilitados e com prótese adequada. As três categorias seguintes indicam indivíduos que necessitam de serviços de reabilitação protética, com diferentes graus de necessidade.

Nesse sentido, considerou-se que essa variável dependente pode ser interpretada como de natureza ordinal. Para fins de padronização, ela foi denominada de “uso e necessidade de prótese total”.

As variáveis independentes em nível individual foram aquelas com coleta similar entre os levantamentos, permitindo comparabilidade. Assim, incluíram-se as variáveis sociodemográficas “sexo” e “raça/cor” e as socioeconômicas “renda” e “escolaridade”.

A variável “sexo” foi mantida com as categorias “masculino” e “feminino”. A raça/cor, autorreferida, foi recategorizada em “branco” e “negro” (junção das categorias “preto” e “pardo”), considerando-se a classificação adotada pelo SB Brasil conforme critérios estabelecidos pelo IBGE^9,13^. Indivíduos classificados como “amarelos” e “indígenas” foram excluídos por conta da baixa frequência e da dificuldade de inclusão nos grupos, além do potencial viés por ser uma variável autorreferida. A categoria “amarela” correspondeu a 1,6% (n=121) em 2010 e 1,3% (n=129) em 2023, enquanto a categoria “indígena” correspondeu a 0,9% (n=72) em 2010 e 0,5% (n=50) em 2023. Desse modo, a baixa frequência poderia gerar vieses importantes de validade externa em relação a essa parcela populacional. Neste estudo, a variável raça/cor foi utilizada como marcador social, sendo considerada como expressão de processos históricos e estruturais de desigualdade.

a “renda familiar” correspondeu ao total recebido por todos os membros no mês anterior à pesquisa. Em 2023, a variável foi registrada como o valor específico em reais (R$), enquanto em 2010 foi categorizada em sete classes (de “até R$ 250” a “R$ 9.500 e mais”). Para a padronização, a renda foi convertida em salários-mínimos, com base no valor atual no momento de cada pesquisa. De acordo com informações do governo brasileiro, o salário-mínimo em 2010 era de R$ 510 e em 2023 de R$ 1.32015. Essa equiparação permitiu a aproximação entre os dados coletados e, por fim, a renda foi agrupada em quatro categorias, a saber: “menor que 1 SM”; “entre 1 e 2 SM”, “entre 3 a 5 SM” e “maior que 5 SM”.

a “escolaridade” foi avaliada em anos de estudo (0 a 16 anos) da mesma forma em ambos os levantamentos, sendo mantida em sua forma original. Para algumas análises, foi dicotomizada com base na mediana de 2023, resultando em “baixa escolaridade” (<8 anos) e “alta escolaridade” (≥8 anos). Para a época em que essa população (65 a 74 anos) teve acesso ao ensino formal, estas categorias correspondem, respectivamente, ao primeiro grau incompleto e o primeiro grau completo ou mais.

Para avaliar o efeito da interseccionalidade, foi realizada uma análise por meio da combinação das variáveis “sexo”, “raça/cor” e “escolaridade”. A inclusão da variável “renda” foi testada, porém resultou em número considerável de valores perdidos.

A combinação dessas três variáveis dicotômicas resultou numa variável com oito categorias, analisada em relação ao pior desfecho, no sentido de verificar sua distribuição e observar um gradiente de desigualdade, conforme a seguinte ordem (da pior para a melhor situação):

1. Homem negro com baixa escolaridade;
2. Mulher negra com baixa escolaridade;
3. Homem branco com baixa escolaridade;
4. Homem negro com alta escolaridade;
5. Mulher branca com baixa escolaridade;
6. Mulher negra com alta escolaridade;
7. Homem branco com alta escolaridade;
8. Mulher branca com alta escolaridade.

Essa classificação foi estabelecida *a priori*, considerando-se que, para o caso das necessidades de prótese total em idosos, sexo masculino, raça/cor negra e baixa escolaridade são importantes fatores de exposição. Essa ordenação não deve ser aplicada em outros contextos nem para outras variáveis.

Finalmente, as regiões do Brasil (Norte, Nordeste, Centro-Oeste, Sudeste e Sul) foram consideradas como variável de contexto por refletirem diferenças socioeconômicas, demográficas e de organização dos serviços de saúde^16^.

### Análise estatística

A análise descritiva foi realizada com base na distribuição proporcional dos desfechos, considerando-se os arranjos entre a variável dependente e as variáveis independentes. Além disso, a região foi incluída como variável de contexto, com o objetivo de identificar diferenças regionais. Todas as análises consideram os dados para os anos de 2010 e de 2023.

Para mensurar a desigualdade na distribuição do desfecho, utilizou-se o Índice de Concentração (IC), calculado de forma similar ao índice de Gini, com base na curva de Lorenz^17^. Esse índice compara a distribuição cumulativa proporcional de uma variável socioeconômica com o mesmo tipo de distribuição de dado desfecho. Valores iguais possuem uma linha reta e indicam ausência de variação (IC=0), enquanto valores entre -1 e 1 indicam diferentes níveis de desigualdade, sendo os extremos correspondentes à condição máxima. Com a ordenação das variáveis de desfecho da melhor para a pior situação e das variáveis de exposição da pior para a melhor situação, valores negativos do IC representam desigualdade^18^.

Nas análises, foi considerado o desenho complexo da amostragem, com a inclusão dos pesos e do efeito de delineamento para a produção das estimativas e para o cálculo do IC. Para este último, foi utilizado o pacote para o *software* Stata desenvolvido pelo Centro Internacional de Equidade em Saúde da Universidade Federal de Pelotas, disponível no endereço https://equidade.org/pt/home.

### Declaração de disponibilidade de dados

Os autores utilizaram dados públicos do SBBrasil para elaboração do estudo que estão disponíveis no site: https://www.gov.br/saude/pt-br/composicao/saps/brasil-sorridente/sb-brasil/dados

## RESULTADOS

A amostra resultante foi, portanto, de 4.462 em 2010 e de 5.222 em 2023. Conforme descrito na seção de método, foram excluídos os indivíduos classificados com a raça/cor “amarela” e “indígena” e, como foram consideradas apenas as necessidades de prótese total, indivíduos com outras possibilidades de reabilitação, como próteses parciais, não foram incluídos na análise.

A análise descritiva da variável de desfecho em relação à variável de contexto (região) e as variáveis individuais está disponível nas Tabelas 1 a 3, com estimativas percentuais e respectivos intervalos de confiança para cada ano estudado. A análise do uso e necessidade de prótese entre pessoas idosas brasileiras revela mudanças importantes entre 2010 e 2023 e esclarece padrões persistentes de desigualdades regionais, raciais e socioeconômicos.

**Tabela 1:** Distribuição de frequências e respectivos intervalos de confiança para a necessidade de prótese total em pessoas idosas (65 a 74 anos), de acordo com o ano e região. Brasil, 2025.

	Ano	2010		2023	
		n	Est. (IC95%)	n	Est. (IC95%)
Norte	Não usa e não necessita prótese em nenhuma arcada	14	1,2 (0,5–2,5)	51	3,5 (2,1–5,8)
	Reabilitado PT em ambas as arcadas	224	18,6 (14,3–23,7)	274	22,3 (16,5–29,3)
	Necessita PT superior e/ou inferior por substituição	488	42,2 (37,2–47,4)	629	44,0 (37–51,4)
	Necessita de PT superior ou inferior por edentulismo	275	23,0 (18,2–28,6)	205	20,1 (16,5–24,2)
	Necessita de PT em ambas as arcadas por edentulismo	129	15,0 (11,5–19,4)	117	10,0 (7,5–13,3)
Nordeste	Não usa e não necessita prótese em nenhuma arcada	29	1,6 (0,9–2,6)	89	4,0 (2,1–7,5)
	Reabilitado PT em ambas as arcadas	316	17,9 (14,9–21,3)	276	13,1 (9,6–17,5)
	Necessita PT superior e/ou inferior por substituição	480	44 (39,1–49)	814	47,4 (41,9–53,0)
	Necessita de PT superior ou inferior por edentulismo	268	21,7 (18,4–25,4)	327	22,2 (18,7–26,2)
	Necessita de PT em ambas as arcadas por edentulismo	162	14,9 (11,5–19)	190	13,3 (9,8–17,8)
Sudeste	Não usa e não necessita prótese em nenhuma arcada	43	2,3 (1,2–4,2)	128	13,9 (9,1–20,7)
	Reabilitado PT em ambas as arcadas	243	31,9 (24,3–40,7)	144	18,3 (13,7–24,0)
	Necessita PT superior e/ou inferior por substituição	295	38,6 (30,9–46,9)	365	49,7 (43,2–56,2)
	Necessita de PT superior ou inferior por edentulismo	115	15,2 (10,5–21,4)	94	10,5 (7,3–14,9)
	Necessita de PT em ambas as arcadas por edentulismo	70	12,1 (8,2–17,3)	52	7,6 (4,5–12,5)
Sul	Não usa e não necessita prótese em nenhuma arcada	37	3,8 (2,1–6,8)	95	9,1 (5,7–14,3)
	Reabilitado PT em ambas as arcadas	233	40,6 (32,5–49,1)	198	33,6 (27,4–40,4)
	Necessita PT superior e/ou inferior por substituição	205	36,5 (26,7–47,5)	291	43,9 (36–52,0)
	Necessita de PT superior ou inferior por edentulismo	85	14,2 (9,7–20,3)	78	8,3 (5,5–12,5)
	Necessita de PT em ambas as arcadas por edentulismo	35	5,0 (2,8–8,7)	30	5,1 (2,8–9,3)
C. Oeste	Não usa e não necessita prótese em nenhuma arcada	17	1,7 (0,8–3,3)	60	6,8 (4,5–10,3)
	Reabilitado PT em ambas as arcadas	178	22,8 (17,6–28,9)	139	18,6 (12,9–26,1)
	Necessita PT superior e/ou inferior por substituição	338	44,5 (37,5–51,7)	417	56,2 (48,7–63,3)
	Necessita de PT superior ou inferior por edentulismo	113	18,4 (14,9–22,5)	97	12 (8,8–16,1)
	Necessita de PT em ambas as arcadas por edentulismo	70	12,7 (8,7–18,2)	62	6,4 (4,1–10)
Brasil	Não usa e não necessita prótese em nenhuma arcada	140	11,9 (7,2–19,0)	423	88,1 (81,0–92,8)
	Reabilitado PT em ambas as arcadas	1.194	46,2 (37,8–54,8)	1.031	53,8 (45,2–62,2)
	Necessita PT superior e/ou inferior por substituição	1.806	30,9 (24,5–38,0)	2.516	69,1 (62,0–75,5)
	Necessita de PT superior ou inferior por edentulismo	856	39,1 (31,2–47,7)	801	60,9 (52,3–68,8)
	Necessita de PT em ambas as arcadas por edentulismo	466	41,7 (31,8–52,2)	451	58,3 (47,8–68,2)

PT: Prótese Total; IC95%: Intervalo de Confiança de 95%; Est.: Estimativa; n: Tamanho da amostra não ponderada.

Fonte: Sobre dados do SB Brasil 2010 e 2023.

**Tabela 2:** Distribuição de frequências e respectivos intervalos de confiança para a necessidade de prótese total em pessoas idosas (65 a 74 anos), de acordo com o ano e variáveis sociodemográficas e econômicas. Brasil, 2025.

Variável	2010				2023			
	n	Est. (%)	IC95%		n	Est. (%)	IC95%	
			LI	LS			LI	LS
Sexo								
Masculino								
Não usa e não necessita prótese em nenhuma arcada	58	3,5	2,1	5,9	161	9,6	6,6	13,8
Reabilitado PT em ambas as arcadas	399	26,2	20,9	32,4	388	19,8	15,6	24,8
Necessita PT superior e/ou inferior por substituição	560	36,4	29,4	44,0	852	45,8	40,5	51,1
Necessita de PT superior ou inferior por edentulismo	311	16,6	11,5	23,4	307	13,4	10,7	16,7
Necessita de PT em ambas as arcadas por edentulismo	230	17,3	12,9	22,8	236	11,5	8,6	15,1
Feminino								
Não usa e não necessita prótese em nenhuma arcada	82	1,7	1,0	2,9	262	9,4	6,7	13,1
Reabilitado PT em ambas as arcadas	795	33,1	27,0	39,8	643	19,4	16,0	23,3
Necessita PT superior e/ou inferior por substituição	1246	41,0	35,0	47,4	1664	49,9	45,2	54,6
Necessita de PT superior ou inferior por edentulismo	545	16,1	12,8	20,0	494	14,2	11,7	17,0
Necessita de PT em ambas as arcadas por edentulismo	236	8,1	5,8	11,2	215	7,1	5,1	9,8
Raça/cor autorreferida								
Branco								
Não usa e não necessita prótese em nenhuma arcada	99	3,6	2,3	5,6	236	15,0	10,5	21,1
Reabilitado PT em ambas as arcadas	624	31,7	25,2	39,1	431	23,7	19,7	28,4
Necessita PT superior e/ou inferior por substituição	813	43,3	36,3	50,7	867	46,6	42,0	51,3
Necessita de PT superior ou inferior por edentulismo	326	14,7	11,3	18,9	219	9,6	7,0	13,0
Necessita de PT em ambas as arcadas por edentulismo	145	6,7	4,8	9,2	113	5,0	3,3	7,5
Negro								
Não usa e não necessita prótese em nenhuma arcada	40	0,9	0,5	1,5	181	4,7	3,3	6,6
Reabilitado PT em ambas as arcadas	537	28,3	22,2	35,2	572	15,8	13,0	19,2
Necessita PT superior e/ou inferior por substituição	938	35,1	28,4	42,5	1555	49,3	44,5	54,2
Necessita de PT superior ou inferior por edentulismo	512	18,4	13,7	24,2	551	17,8	15,2	20,8
Necessita de PT em ambas as arcadas por edentulismo	305	17,4	12,6	23,5	324	12,3	9,2	16,3
Escolaridade (anos de estudo)								
8 anos ou mais								
Não usa e não necessita prótese em nenhuma arcada	98	8,6	4,9	14,8	340	21,1	16,3	26,8
Reabilitado PT em ambas as arcadas	266	32,7	24,0	42,7	496	20,1	16,5	24,2
Necessita PT superior e/ou inferior por substituição	376	47,5	36,8	58,5	1040	46,4	41,6	51,4
Necessita de PT superior ou inferior por edentulismo	94	6,4	4,2	9,7	222	9,0	6,5	12,4
Necessita de PT em ambas as arcadas por edentulismo	41	4,8	2,0	10,8	89	3,4	2,1	5,4
Até 7 anos								
Não usa e não necessita prótese em nenhuma arcada	39	1,0	0,5	2,1	69	2,0	1,1	3,7
Reabilitado PT em ambas as arcadas	900	30,2	24,6	36,5	515	19,9	16,4	23,8
Necessita PT superior e/ou inferior por substituição	1385	38,3	32,7	44,3	1422	50,3	45,3	55,2
Necessita de PT superior ou inferior por edentulismo	735	18,3	14,4	23,0	550	16,8	14,2	19,8
Necessita de PT em ambas as arcadas por edentulismo	401	12,1	9,3	15,7	332	11,0	8,4	14,4
Renda média mensal familiar								
3 ou mais S.M.								
Não usa e não necessita prótese em nenhuma arcada	96	5,2	2,8	9,4	169	29,3	19,5	41,4
Reabilitado PT em ambas as arcadas	384	38,4	30,0	47,6	155	22,2	16,2	29,5
Necessita PT superior e/ou inferior por substituição	489	40,7	31,6	50,6	259	42,7	32,7	53,4
Necessita de PT superior ou inferior por edentulismo	160	7,2	4,8	10,8	49	3,9	2,1	7,2
Necessita de PT em ambas as arcadas por edentulismo	66	8,4	4,8	14,3	25	1,9	0,8	4,3
Até 2 S.M.								
Não usa e não necessita prótese em nenhuma arcada	41	1,4	0,7	2,6	140	5,8	3,8	8,7
Reabilitado PT em ambas as arcadas	771	28,3	22,9	34,3	566	19,5	15,9	23,8
Necessita PT superior e/ou inferior por substituição	1249	39,0	33,2	45,1	1558	47,8	43,3	52,3
Necessita de PT superior ou inferior por edentulismo	666	19,0	15,2	23,3	548	16,7	13,8	20,0
Necessita de PT em ambas as arcadas por edentulismo	387	12,4	9,4	16,3	275	10,2	7,8	13,3

PT: Prótese Total; S.M: Salário-Mínimo; IC95%: Intervalo de Confiança de 95%; Est.: Estimativa; LI: Limite Inferior; LS: Limite Superior.

Fonte: Sobre dados do SB Brasil 2010 e 2023.

**Tabela 3:** Distribuição de frequências e respectivos intervalos de confiança para a necessidade de prótese total em pessoas idosas (65 a 74 anos), de acordo com o ano e categorias de interseccionalidade (sexo, raça/cor e escolaridade). Brasil, 2025.

Variável		Homem negro com baixa escolaridade	Mulher negra com baixa escolaridade	Homem branco com baixa escolaridade	Homem negro com alta escolaridade	Mulher branca com baixa escolaridade	Mulher negra com alta escolaridade	Homem branco com alta escolaridade	Mulher branca com alta escolaridade
Não usa e não necessita prótese em nenhuma arcada	Est. (%)	1,0	0,2	2,0	7,0	1,4	1,3	13,1	9,7
	IC95%	(0,4–2,4)	(0,01–0,50)	(0,8–5,0)	(2,6–18,0)	(0,4–5,0)	(0,5–3,3)	(5,2–29,4)	(5,5–16,3)
	n	13	8	8	9	10	8	27	53
Reabilitado PT em ambas as arcadas	Est. (%)	24,2	32,9	23,2	26,8	32,9	15,3	33,4	41,6
	IC95%	(15,5–35,8)	(25,6–41,1)	(16,8–31,2)	(12,5–48,6)	(24,2–42,8)	(7,2–29,8)	(19,7–50,7)	(30,2–54,0)
	n	143	293	130	35	308	51	70	104
Necessita PT superior e/ou inferior por substituição	Est. (%)	32,0	32,7	40,1	41,8	45,9	67,9	41,8	44,6
	IC95%	(21,8–44,3)	(25,6–40,7)	(30,4–50,6)	(22,3–64,3)	(35,9–56,2)	(46,1–84)	(26,4–59,1)	(32,2–57,7)
	n	215	515	190	58	422	121	63	126
Necessita de PT superior ou inferior por edentulismo	Est. (%)	19,2	20,5	19,1	7,3	16,2	7,9	9,2	3,0
	IC95%	(9,8–34,3)	(15,1–27,3)	(12,6–27,9)	(3,4–14,8)	(11,5–22,2)	(3,5–16,9)	(4,8–16,8)	(0,9–9,3)
	n	160	291	95	21	178	25	21	24
Necessita de PT em ambas as arcadas por edentulismo	Est. (%)	23,6	13,7	15,5	17,0	3,8	7,50	2,50	1,20
	IC95%	(15,2–34,6)	(9,6–19,3)	(10,3–22,6)	(4,8–45,7)	(2,2–6,3)	(1,1–36,7)	(0,8–7,5)	(0,5–3)
	n	121	143	68	16	55	7	9	8
2023									
Não usa e não necessita prótese em nenhuma arcada	Est. (%)	1,1	1,4	2,2	10,3	3,5	12,3	28,5	29,6
	IC95%	(0,5–2,2)	(0,6–3,1)	(0,7–6,4)	(6,5–16)	(1,2–9,7)	(7,5–19,6)	(18,6–41,2)	(21,2–39,8)
	n	14	31	8	52	15	76	83	124
Reabilitado PT em ambas as arcadas	Est. (%)	14,0	16,3	22,9	20,6	26,5	16,1	25,5	20,3
	IC95%	(9,1–21,2)	(11,8–22,1)	(14,4–34,3)	(13,6–29,9)	(20,1–34,1)	(11,3–22,3)	(16,1–37,9)	(12,8–30,6)
	n	107	194	66	104	129	157	92	134
Necessita PT superior e/ou inferior por substituição	Est. (%)	45,7	49,2	55,9	48,4	51,9	59,6	34,5	41,4
	IC95%	(37,1–54,5)	(42,0–56,4)	(42,9–68,2)	(40,0–56,9)	(44,0–59,8)	(49,7–68,7)	(25,6–44,6)	(33,1–50,2)
	n	316	585	146	208	325	417	133	249
Necessita de PT superior ou inferior por edentulismo	Est. (%)	19,5	21,1	9,9	14,3	13,3	9,2	7,2	6,9
	IC95%	(14,0–26,6)	(16,6–26,5)	(5,6–17,0)	(9,6–20,9)	(8,8–19,7)	(6,1–13,7)	(2,2–21,5)	(4–11,6)
	n	146	244	50	55	89	81	32	45
Necessita de PT em ambas as arcadas por edentulismo	Est. (%)	19,7	12,0	9,0	6,4	4,7	2,9	4,3	1,8
	IC95%	(14,1–26,9)	(7,7–18,2)	(4,3–17,9)	(2,9–13,4)	(2,6–8,4)	(1,4–5,6)	(1,4–12,4)	(0,8–4,1)
	n	137	111	36	27	35	27	17	18

PT: Prótese Total; IC95%: Intervalo de Confiança de 95%; Est.: Estimativa.

Fonte: Sobre dados do SB Brasil 2010 e 2023.

Entre 2010 e 2023, houve melhora nas condições de reabilitação das pessoas idosas brasileiras, com pequeno aumento da proporção daqueles que não usam e não necessitam de prótese dentária, especialmente no sul e sudeste. Em 2010, predominavam indivíduos que necessitavam de alguma prótese total por substituição, enquanto a proporção de ausência de necessidade era baixa (até 3,8%). Em 2023, embora a necessidade de substituição da reabilitação ainda predomine, observa-se a melhoria sutil no desfecho positivo (até 13,9%) e redução da necessidade de prótese por edentulismo. Apesar disso, o cenário de necessidade por qualquer motivo ainda figura em torno da porcentagem de 90%.

As desigualdades observadas nas análises por sexo, raça, escolaridade e renda perduraram ao longo do período. Mulheres apresentaram maior melhora na preservação dentária, enquanto homens mantiveram o protagonismo nas necessidades não atendidas, apesar da maior redução por edentulismo. Entre os grupos raciais, todos avançaram, mas pessoas idosas negras seguem em desvantagem, com menor proporção de indivíduos livres de necessidade em ambos os anos e menor redução de necessidade por edentulismo, refletindo desigualdades tanto na qualidade e durabilidade das reabilitações quanto no acesso aos serviços.

Os gradientes de renda e escolaridade têm uma relação diretamente proporcional, em que, quanto menor o nível educacional e a renda, piores os indicadores de saúde bucal. Em 2023, 29,3% das pessoas idosas de maior renda não necessitavam de prótese contra apenas 5,8% entre os de menor renda. Apesar da melhoria na comparação com 2010, a desigualdade social parece superar os impactos positivos alcançados. Assim, ainda que o panorama geral revele avanços, os resultados demonstram que as melhorias não foram homogêneas, algo que fica ainda mais claro da perspectiva da interseccionalidade.

A análise interseccional do uso e necessidade de próteses evidencia o agravamento das desigualdades sociais e raciais entre 2010 e 2023. O desfecho negativo tornou-se mais concentrado entre pessoas idosas com condições cumulativas de: menor escolaridade, sexo masculino e raça não branca, revelando que os progressos advindos das políticas com escopo de reabilitação bucal não foram suficientes para reduzir as iniquidades históricas. Ao contrário, as barreiras estruturais de acesso e cuidado parecem ter aumentado, potencializando padrões existentes de exclusão no campo.

A Figura 1 apresenta as curvas de concentração (curva de Lorenz) do uso e necessidade de prótese segundo quatro variáveis selecionadas e, na Figura 2, os valores do IC e respectivos intervalos de confiança para as variáveis estudadas, por ano. As curvas ilustram que, entre 2010 e 2023, renda, escolaridade, raça e a variável da interseccionalidade permaneceram determinantes centrais das desigualdades, com agravamento ao longo do período.

**Figura 1:** Curvas de concentração para o uso e necessidade de prótese em relação às variáveis sociodemográficas e econômicas. A linha verde representa a linha de igualdade perfeita das curvas de concentração. Brasil, 2025.

Fonte: Sobre dados do SB Brasil 2010 e 2023.

**Figura 2:** Valores e respectivos intervalos de confiança para o Índice de Concentração para o uso e necessidade de prótese, de acordo com as variáveis independentes utilizadas. Brasil, 2025.

Fonte: Sobre dados do SB Brasil 2010 e 2023.

Embora se observem melhoras relativas em alguns grupos, as curvas e os valores do CI mostram que o pior desfecho continua concentrado entre as pessoas idosas em maior vulnerabilidade social. As desigualdades associadas à renda e à escolaridade apresentaram os maiores níveis, revelando que o acesso e a qualidade da reabilitação protética seguem fortemente condicionados por fatores estruturais. Quando combinados os determinantes sexo, raça e escolaridade, o índice atinge os valores mais elevados, reforçando a dimensão interseccional.

Estes achados atestam que os avanços nas políticas públicas de reabilitação bucal e as melhorias observadas entre os grupos sociodemográficos ocorreram de forma desigual, com melhores indicadores concentrados entre grupos socialmente favorecidos e lacunas persistentes no cuidado com as pessoas idosas vulnerabilizadas.

## DISCUSSÃO

Os avanços observados não ocorreram de forma homogênea, com as desigualdades individuais mantidas ou, em alguns casos, agravadas. Observou-se que a diminuição da perda dentária e o acesso à reabilitação protética permanecem condicionados por fatores socialmente estruturados. Assim, apesar da melhora na saúde bucal, as desigualdades relacionadas à renda, raça/cor e escolaridade refletem uma tendência pró-ricos de acesso, o que pode ser influenciado por uma articulação ainda limitada entre macropolítica e micropolítica^19,20^.

Fatores regionais de gênero, raça/cor, escolaridade e renda continuam associados ao acesso à reabilitação odontológica, refletindo problemas marcados por dificuldades no contato prévio com ações preventivas e de promoção da saúde. Esse contexto é compatível com a persistência de processos estruturais que organizam o acesso aos serviços de saúde, incluindo aqueles relacionados ao racismo estrutural^21^, os quais podem ser mais danosos às pessoas idosas analisadas, que vivenciaram fases da vida sem um sistema universal de saúde e, consequentemente, apresentam maior exposição ao gradiente social.

O aumento da proporção de pessoas idosas sem necessidade de prótese entre os indivíduos de maior renda e escolaridade pode indicar limitações das políticas de saúde bucal na promoção da equidade, além de possivelmente refletir maior acesso a serviços privados, cujo padrão é mais frequente nas Regiões Sul e Sudeste^5^, que concentram melhores cenários socioeconômicos. Essa distribuição remete ao conceito do acesso, segundo o qual a utilização dos serviços odontológicos reabilitadores não depende apenas da disponibilidade, mas também de fatores sociais que condicionam o seu uso.

A análise desses determinantes da perspectiva da interseccionalidade mostrou-se relevante, uma vez que a sobreposição de marcadores sociais produziu um gradiente de desigualdade, no qual diferentes combinações estiveram associadas a piores desfechos. Esses achados sugerem que os efeitos das desigualdades não atuam de forma isolada, mas de maneira cumulativa. Ainda que não seja possível estabelecer causalidade, esses padrões corroboram achados de outras investigações que apontam a persistência de barreiras estruturais no acesso aos serviços de saúde bucal e piores condições de saúde em determinados grupos populacionais^22–26^.

Os achados indicam menor proporção de indivíduos negros reabilitados ou sem necessidade de prótese e maior demanda por substituição e edentulismo. Esses resultados evidenciam desigualdades na distribuição do desfecho que, embora associadas a fatores socioeconômicos, não se explicam exclusivamente por eles, demonstrando desigualdades complexas que extrapolam o sistema de saúde. Neste estudo, a variável raça/cor é compreendida como um marcador social que expressa processos históricos e estruturais de desigualdade. Tais diferenças se relacionam com o contexto de desigualdades estruturais, incluindo o racismo^26–28^.

Em contrapartida, análises comparativas entre 2003 e 2010 apontam melhorias mais expressivas, em um período marcado pela ampliação de políticas sociais, que podem ter interagido com os desfechos observados, sugerindo uma possível relação dose-resposta.

A expansão da cobertura da atenção básica e a consolidação da PNSB foram fundamentais para ampliar o acesso aos serviços odontológicos, incluindo as ações das equipes de saúde bucal (eSB) e assistência especializada ofertada pelos CEOs e LRPDs^29^. Contudo, essas iniciativas ainda se mostram insuficientes para reduzir as iniquidades e saúde, uma vez que a permanência de proporções próximas a 70% de pessoas idosas com necessidade de prótese, seja por edentulismo, seja por substituição, demonstra que a reabilitação protética permanece como demanda reprimida^5,30^.

As iniquidades na necessidade de prótese dentária por substituição e no edentulismo evidenciam desigualdades estruturais relacionadas ao acesso, à qualidade e à continuidade do cuidado odontológico^31^. Nesse sentido, torna-se necessário ultrapassar estratégias centradas apenas na ampliação da oferta, integrando ações de reabilitação, promoção e prevenção, alinhadas aos determinantes sociais do processo saúde-doença. Isso requer prioridades sustentáveis, financiamento adequado e fortalecimento da Atenção Primária como eixo organizador da rede de atenção à saúde bucal, com vistas à ampliação do acesso equânime e universal^32,33^.

Como limitações, sabe-se que a acurácia do IC pode ter sido parcialmente prejudicada em função da utilização de uma variável ordinal, porém percebe-se que o IC evidenciou cenários de desigualdade que ressaltam seu poder discriminatório. A redução de categorias interseccionais complexas a uma escala ordinal limita a aproximação da quantificação das dimensões sociais e suas interações na realidade, o que demanda estudos posteriores com estratégias analíticas mais profundas. O caráter transversal do SB Brasil limita as análises à identificação de associações, impedindo inferências causais. Quanto às pessoas amarelas e indígenas, a baixa representatividade categórica no estudo dessa importante parcela da população brasileira justifica sua exclusão para maior parcimônia do modelo estatístico. Como pontos fortes, destacam-se o uso de desenho amostral complexo, com preservação de pesos e representatividade, e a inovação metodológica representada pela construção de uma variável dependente composta, que incorpora diferentes fatores relacionados à necessidade de prótese.

Apesar das melhorias observadas na saúde bucal da população idosa, persiste uma elevada demanda por substituição de próteses inadequadas. O enfrentamento desse problema requer, além da ampliação da cobertura, a implementação de políticas públicas intersetoriais orientadas à equidade territorial, racial e social. Para isso, é fundamental fortalecer a articulação entre micropolíticas e macropolíticas, de modo a promover um cuidado em saúde que considere as vulnerabilidades sociais.

## APROVAÇÃO CEP:

A pesquisa não necessitou de submissão ao CEP por se tratar de dados secundários.
